# Baseline evaluation of infection prevention and control (IPC) in the context of Ebola virus disease (EVD) in nine healthcare facilities in the city of Conakry, Guinea

**DOI:** 10.1186/2047-2994-4-S1-O10

**Published:** 2015-06-16

**Authors:** A Diallo, M Diallo, Y Hyjazi, R Waxman, T Pleah

**Affiliations:** 1 Jhpiego, Conakry, Guinea; 2 Jhpiego, Baltimore, MD, USA

## Introduction

As a means to contribute to the response to the Ebola virus disease (EVD) epidemic and support health system strengthening, Jhpiego, with funding from USAID, committed to strengthening health service capacity in IPC.

## Objectives

Determine IPC performance level in selected health facilities.

Identify gaps in IPC performance.

## Methods

The assessment was conducted in December 2014 and January 2015 in three teaching hospitals, comprising 66 services, and six communal medical centers (CMC). The main assessment tool was based on SBM-R performance standards for IPC. Data collection consisted of observation, interview and document review by a team of two evaluators per facility, for 2-3 days per facility.

## Results

In the three national teaching hospitals, among 66 services, one service achieved 75% of performance standards, and eight achieved none. 33 of the services achieved below 30% of standards, while 17 scored between 31 and 49% and seven were performing at 50-74% of standards. In the 6 CMC, four were performing between 16 and 20% of standards, and two were performing at 35% and 37% respectively. Key reasons for poor performance included: 1) Insufficient knowledge in IPC, 2) lack of IPC materials and equipment, 2) non-observance of IPC procedures and norms, 3) poor management of IPC activities.

## Conclusion

Key interventions included training of healthcare workers, provision of an initial stock of IPC materials, onsite follow-up. These actions will contribute to the improvement of healthcare services, and help to restore community confidence in the public health services.

## Disclosure of interest

None declared.

